# Alterations in the Microbiota of Caged Honeybees in the Presence of *Nosema ceranae* Infection and Related Changes in Functionality

**DOI:** 10.1007/s00248-022-02050-4

**Published:** 2022-07-12

**Authors:** Daniele Alberoni, Diana Di Gioia, Loredana Baffoni

**Affiliations:** Department of Agricultural and Food Sciences, University of Bologna, Viale Fanin 44, 40127 Bologna, Italy

**Keywords:** Lactobacillus, Bifidobacterium, Serratia, Gut microbiota, Honeybees, Nosemosis

## Abstract

Several studies have outlined that changes in the honeybee gut microbial composition may impair important metabolic functions supporting the honeybees’ life. Gut dysbiosis may be caused by diseases like *Nosema ceranae* or by other anthropic, environmental or experimental stressors. The present work contributes to increasing knowledge on the dynamics of the gut microbiome acquisition in caged honeybees, an experimental condition frequently adopted by researchers, with or without infection with *N. ceranae*, and fed with a bacterial mixture to control *N. ceranae* development. Changes of the gut microbiota were elucidated comparing microbial profile of caged and open-field reared honeybees. The absolute abundance of the major gut microbial taxa was studied with both NGS and qPCR approaches, whereas changes in the functionality were based on RAST annotations and manually curated. In general, all caged honeybees showed important changes in the gut microbiota, with $\gamma$-proteobacteria (*Frischella*, *Gilliamella* and *Snodgrassella*) lacking in all caged experimental groups. Caged honeybees infected with *N. ceranae* showed also a strong colonization of environmental taxa like *Citrobacter*, *Cosenzaea* and *Morganella*, as well as possibly pathogenic bacteria such as *Serratia*. The colonization of *Serratia* did not occur in presence of the bacterial mixture. The functionality prediction revealed that environmental bacteria or the supplemented bacterial mixture increased the metabolic potential of the honeybee gut microbiome compared to field and caged controls.

**Supplementary Information:**

The online version contains supplementary material available at 10.1007/s00248-022-02050-4.

## Introduction

Honeybees (*Apis mellifera*) are social insects with a high environmental importance and economic impact on the agricultural sector through pollination service, crop yield increase and hive products [1, 2]. Recently, honeybees’ efficiency in supporting ecosystem services and agriculture has been decreasing due to an increased number of stressors. New emerging diseases, such as nosemosis of type C [3] and chronic bee paralysis virus (CBPV) [4] are easily finding synergies with climate change [5, 6], habitat loss and agrochemicals [7, 8], leading to honeybee decline [9]. The challenge posed to beekeepers, who have to constantly repopulate the loss of colonies, is also a major concern. Among emerging diseases, *N. ceranae* has gained a particular attention because it shows a long asymptomatic period of incubation in the honeybees colonies [10], but it outbreaks with fast and severe symptoms leading to colony collapse. *N. ceranae* is a microsporidian gut parasite evolved with the Asiatic honeybee (*Apis ceranae*). Known since 1996, *N. ceranae* shifted from its original Asiatic host to the European honeybee *A. mellifera*, leading to severe outbreaks of nosemosis type C in Europe and America [11–13]. In the last decades, a number of feed supplements based on natural compounds have been studied as a possible alternative to the use of antibiotics and improve honeybee’ immune response to pests and pathogens and, in general, improve bee health. Examples of such feed supplements are plant extracts like thymol and laurel extracts [14, 15], phytohormones like abscisic acid (ABA) [16, 17] and beneficial bacteria or bacterial secondary metabolites [18–22].

Both feed additives and gut diseases may have an impact on the structure and functionality of the gut microbial community of honeybees that is composed of 8 core bacterial phylotypes present in almost all honeybees (*Bartonella*, *Bifidobacterium*, *Bombilactobacillus*, *Commensalibacter*, *Frischella*, *Gilliamella*, *Lactobacillus*, *Snodgrassella*). These bacteria show specific functions to support the host improving the nutrient digestion, host defense from pathogens, and immune system activation  [23]. Moreover, a number of facultative endosymbiotic bacterial genera (*Apibacter*, *Asaia*, *Arsenophonus*, *Citrobacter*, *Cosenzaea*, *Wolbachia*, *Morganella*, *Pseudomonas* and *Spiroplasma*) may occasionally colonize the honeybee gut [24] but their benefits for the host, if any, have not been fully elucidated. The proportion of the core and non-core bacteria in the honeybees gut is not only affected by larval and adult onthogenic stage  [25], the in-hive functions  [26], the seasonality and habitat  [27], but also by the dietary supplements administered to the bees  [28]. In the work carried out by Baffoni et al.  [21], a daily supplementation of a beneficial bacteria mixture to honeybees significantly decreased *N. ceranae* spore load of naturally infected honeybees in laboratory cage condition, thus showing an antagonistic activity on the gut parasite development, with a mechanism of action hypothesized in feed acidification and immune system stimulant effect on honeybees. However, in the mentioned study, the effect of the administration of selected bacteria as well as of *N. ceranae* infection on the honeybee gut microbiota was not examined. To the best of our knowledge, three research works are available [29–31] on the study of the microbiota alterations of artificially reared honeybees with the concomitant inoculation of the gut parasite *N. ceranae*: Paris et al.  [29], testing the synergy with different pesticides (fipronil, thiamethoxam, and boscalid); Castelli et al. [30], assessing the nutritional potential of two different pollen sources (mono-floral and poly-floral); Zhang et al. [31], focusing on two different sugar diets (isomaltooligosaccharide + sucrose and sucrose only). Powell et al. [32] showed that microbiota acquisition in newborn honeybees may be incomplete if there is a limited or no social transmission with adult honeybees that allows trophallaxis and coprophagy, a condition that particularly affects the $\gamma$-Proteobacteria acquisition. However, the mentioned research works [29–31] showed that there was complete microbiome acquisition in the caged honeybees, allowing to speculate that prior to caging, honeybees had a sufficiently long contact with adult honeybees or hive surface to allow a complete gut colonization.

The present work investigates the gut microbiota of caged honeybees infected or not with *N. ceranae* and upon administration of a bacterial mixture with metagenomic approaches. The microbiota composition of caged honeybees was then compared with that of honeybees collected from colonies in open field. Particular care was used in the selection of newborn honeybees to be caged, to make sure that there was no contact with the hive surface and colony mates, apart from the cell capping. The objective of this approach was to confirm which microbial genera are acquired without a proper social cohesion. Moreover, according to the main microbial taxa detected, a functional prediction of the expression of vitamins, amino acids, and degradation of polysaccharides was attempted. Finally, a new database for taxonomy assignment, specifically focused on honeybee gut microbial species, was designed and tested (referred to as InsectGene database).

## Methods

### Workflow

In order to obtain new strains for the new database implementation and for functional analysis, microbial strains (in particular Lactobacillaceae) were isolated from honeybee guts and characterized through pulsed field gel electrophoresis (PFGE) and whole-genome sequencing (WGS) (Sections “2.2” and “2.3)”. At the same time, honeybee gut samples obtained from the experimental conditions described in Baffoni et al. [21] were analysed for their microbiome composition in qPCR and amplicon-based next-generation sequencing (NGS) (Sections “ 2.4”–“2.6”). Finally, the data obtained from the isolation, WGS, and NGS metagenomic were used for the new database implementation (Section “2.7”) and the gut microbiome functionality prediction (Section “2.8”).

### Microbial Isolation, Lactobacillaceae Strain Typing with PFGE, and 16S rRNA Gene Sequencing

Gut samples of honeybee deriving from the hives of origin of the cage test described in Baffoni et al. [21] were used as a source of new microbial strains. Serial dilutions of the gut content were prepared and plated on de Man Rogosa Sharpe medium (MRS) (Becton Dickinson, Mountain View, CA), containing 0.2% (w/v) sorbic acid (Sigma-Aldrich, Milan, Italy) and 0.1% (w/v) cycloheximide (Sigma-Aldrich). Plates were incubated in anaerobic condition at 35±1 $^\circ C$. Colonies were picked up, re-streaked, and purified on the same medium. Putative isolated Lactobacillaceae strains were typed with PFGE. Lactobacillaceae were grown on MRS with 2% fructose, 0.1% L-cysteine hydrochloride [33] and with 20 mM D-threonine (Sygma-Aldrich, Milan, Italy) to facilitate lysis  [34]. Cells were harvested from 0.5-mL overnight culture, washed once in 500 $\mu$L 10 mM Tris HCl, 1 M NaCl (pH 7.6), and re-suspended in 300 $\mu$L of the same buffer. The suspension was mixed with an equal volume of 2% of PFGE low melting point agarose (Bio-Rad, Segrate, Italy) before solidifying in plugs. Plugs were incubated in a lysis buffer containing mutanolysin 20 units/mL [35] and treated with proteinase K overnight at 55 $^\circ$C. Obtained plugs were restricted overnight with SmaI (New England BioLabs, Hertfordshire, UK). DNA fragments were resolved using a CHEF-DR III pulsed-field system (Bio-Rad Laboratories, Segrate, Italy) at 6 V/cm for 16 h with pulse time ramped from 1 to 20 s. In every gel, a low-range PFG marker (New England BioLabs, Hertfordshire, UK) was used as ladder for gel normalization. After ethidium bromide staining, gel images were digitized using Gel Doc XR+ Gel Documentation System (Bio-Rad, Segrate, Italy). The 16S rRNA gene Sanger sequencing was performed on a selection of strains according to the PFGE results [36].

### Whole-Genome Sequencing and Genome Assembly

Strains of the bacterial mixture administered to honeybees (Section “2.4”) as well as a selection of 6 isolated Lactobacillaceae strains chosen after the PFGE results (Table S1) were subjected to WGS. Genomic DNA was extracted using the Promega Wizard Genomic DNA extraction kit (Promega, Madison, USA). DNA concentration and purity were determined by measuring the absorbance at 260 and 280 nm. The extracted DNA was stored at −20 $^\circ$C until further analysis. WGS was performed by MicrobesNG (University of Birmingham, Birmingham, UK) on Illumina HiSeq, and obtained sequences assembled with SPAdes [37].

### Cage Test: Sample Collection and Processing

**Fig. 1:**
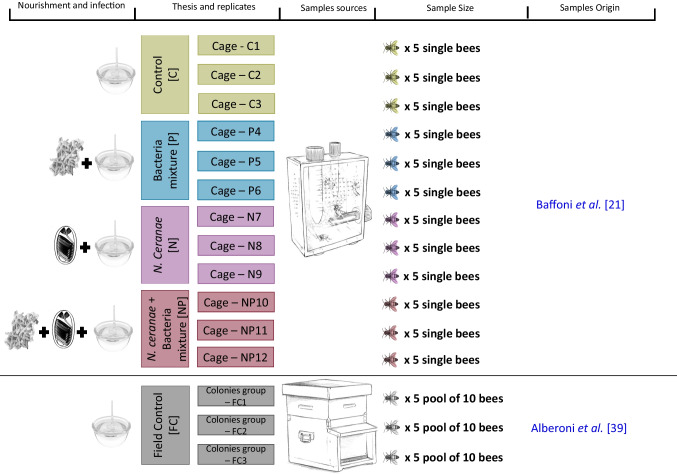
**Graphical representation of the experimental design.** Experimental conditions were [C] honeybees fed with sugar syrup; [P] honeybees fed with sugar syrup enriched with BB mixture; [N] honeybees fed with sugar syrup and infected with *N. ceranae*; [NP] honeybees fed with sugar syrup enriched with BB mixture, and infected with *N. ceranae*; [FC] honeybees reared in field condition

Samples were collected during a previously described cage test study  [21] briefly summarized here. A brood frame containing 13-day-old honeybee pupae was picked from an experimental apiary (Bologna, Italy) and incubated at 33 $^\circ$C and 65% relative humidity (RH). Just before enclosure, honeybees were gently extracted with tweezers from their wax cells, in order to prevent contact with the wax frame surface or with other emerging honeybees and inserted in the experimental cages. *N. ceranae* spores were collected from an infected apiary and purified with a 95% Percoll solution  [21] and quantified in a cell counting chamber [38]. The beneficial bacterial mixture composed of *Bifidobacterium asteroides* C3 (DSM 20431), *B. coryneforme* C155 (LMG 30569), *B. indicum* C449, *Apilactobacillus kunkeei* Dan39 (LMG 30566), *Lactiplantibacillus plantarum* Dan91 (LMG 30567) and *Lactobacillus johnsonii * Dan92 (LMG 30568), as already described [21], was administered at the concentration of 10^6^–10^7^ cfu/mL of sugar syrup. Four theses were developed, as shown in Fig. 1, each replicated three times: honeybees fed with sugar syrup as control [C]; honeybees fed with sugar syrup enriched with the beneficial bacterial mixture referred to as Probiotics [P]; honeybees fed with sugar syrup and infected with 10,000 spores of *N. ceranae* at the 5^th^ day of life [N]; honeybees fed with sugar syrup enriched with the beneficial bacterial mixture, and infected with 10,000 spores of *N. ceranae* at the 5^th^ day of life [NP]. Sugar syrup was administered daily. At day 9, 120 honeybees (30 for each experimental condition) were sacrificed after anesthetization, the gut (midgut, ileum, and hindgut) was excised and DNA extracted with the ZR Tissue and Insect DNA MicroPrep  [21]. The extracted DNA was quantified with Qubit^TM^ dsDNA HS Assay Kit (Thermo Fisher, Milan, Italy) and used for qPCR analysis (Section “2.5”) and 16S rRNA gene sequencing via NGS (Section “2.6”). Finally, to compare the gut microbiota of caged honeybees with that of in-field conditions, sequencing data of the honeybee gut microbiome of 15 different samples were retrieved from a previous open-field study  [39] and used for comparative analyses. These samples are referred to as Field Control [FC] (samples list is presented in Table S2). The age of sampled honeybees was approximately the same of this study.

### Quantitative PCR

The total number of bacteria (Eubacteria) in the honeybee gut content was determined at day 9 (as reported in Section “2.4”) via qPCR assay according to Alberoni et al. [39], using the primers Eub338-F 3’ACTCCTACGGGAGGCAGCAG-5’ and Eub518-R 3’ATTACCGCGGCTGCTGG-5’. After analysing the NGS data (see “Results” below), the genus *Serratia* was considered worthy of absolute quantification using qPCR. The quorum sensing LuxS gene was selected as molecular marker for *Serratia* quantification, using primers luxS1: 3’-TGCCTGGAAAGCGGCGATGG-5’ and luxS2: 3’-CGCCAGCTCGTCGTTGTGGT-5’ [40]. Briefly, standard curves were constructed using PCR products of target genes, purified, and serially diluted to obtain standards ranging from 10^4^ to 10^8^ copies. Quantification was performed using Fast SYBR Green Master Mix (Applied Biosystems) on a 10 $\upmu$L reaction.

### 16S rRNA Based Next Generation Sequencing and Bioinformatics Analysis

NGS was performed on DNA from 15 honeybees per experimental condition (5 deriving from each cage as depicted in Fig. 1) randomly chosen among the whole set of samples collected in Baffoni et al. [21]. Therefore, a total of 60 samples were analysed via NGS in this work. The V3–V4 regions of 16S rRNA gene were amplified with primers Pro341F and Pro805R (Takahashi et al. [41]), barcoded and sequenced on the MiSeq Illumina platform 2x300 bp V3 chemistry according to the protocol of Baffoni et al. [24]. Raw reads were analysed with Qiime II [42], chimera checked with Userach61 [43], and obtained representative OTUs blasted against SILVA database v132 [44] and the new *Apoidea*-specific database. OTUs with less than 0.1% abundance were removed and bar charts generated.

### Building a Fast Annotation 16S rRNA Gene Database Specific for Honeybees

A new database was developed, named InsectGene, containing only 16S rRNA gene sequences of microorganisms from *Apoidea*. The 16S rRNA gene sequences for the new database were obtained from the following sources: (i) previous analyses obtained with SILVA database v132 on the same sample set considered within this work; (ii) existing literature data on honeybee metagenomic analyses; (iii) existing literature of novel commensal species from honeybees; (iv) bacteria isolation on selected growth media in the past years from guts of Hymenoptera within DISTAL (Table S3 and  Section “2.1” of this work). Full-length sequences of 16S rRNA genes from type strains were retrieved from NCBI according to Table S4, but when full-length sequences were unavailable, also partial sequences were included, if considered relevant. All taxonomic assignments were manually curated based on NCBI taxonomy database, with the updated nomenclature [45]. Taxonomic classifications were formatted according to the GreenGene database structure and made available in comma-separated value (CSV) file. The multiple sequence alignment FASTA file was obtained with MEGA 7, maximum likelihood phylogeny. Finally, the database was validated, both comparing the taxonomic assignment obtained with SILVA v132 and the new database, and also manually verifying the accurateness of taxonomic assignments of random OTUs on NCBI blast.

### Metagenome Functionality Prediction

NGS of 16S rRNA marker gene was used to obtain inference of the functional profile of the microbial communities for the different experimental conditions. Annotation of functionality of taxa evidenced in the different samples was carried out with RAST (SEED Viewer version 2.0) [46, 47] on (i) fully sequenced genomes of type strain bacteria retrieved from NCBI GenBank, according to the identified taxa with 16S rRNA gene metagenome (see Table S5); (ii) strains isolated from the same honeybees in this experiment (Table S1); and (iii) on the sequencing of strains administered as feed supplement. In this work, particular attention was given to the analysis of metabolic pathways for the synthesis of vitamins, amino acid, carbohydrate degradation and pathways linked to lignin, cellulose, chitin, and murein digestion. Functional maps of specific metabolic pathways were analysed relying on KEGG orthology database [48] linked with RAST web service. Each microbial strain was checked for presence/absence of the selected relevant pathways, the degree of pathway completeness and the copy-number of genes available within the same genomes. When more than one microbial strain per species was used, the target metabolic activity is expressed as average of the strains considered. Results were plotted in a Dot-Plot Chart and expressed in a scale 0–5 according to the potential detected. Finally, relative genetic potential of each representative taxon was multiplied with the absolute abundance of taxa detected in the sampled honeybees and non-parametric statistics applied.

### Statistical Analysis

Statistical analysis of qPCR and NGS data was carried out according to Alberoni et al. [49]; briefly, analysis was performed with R software considering the data normality and homoscedasticity. GLM procedure was used for non-normal data with normal distribution of residuals and Kruskal-Wallis (Dunn test post hoc analysis) test for non-normal data. Moreover, Bonferroni’s correction was applied, considering 9 experimental conditions comparisons ([C] *vs* [FC], [P], [N] and [NP]; [FC] *vs* [P], [N] and [NP]; [N] *vs* [NP] and [P] *vs* [NP]). PCA analyses were performed with R packages FactoMineR [50] and factoextra [51], taking into consideration 12 taxa at genus level and 15 taxa at species level. Statistical analysis on the metabolic potential of the different experimental conditions was computed as well with GLM as previously described, coupled with an analysis of biological relevance (CramerV) carried out with R package ‘rcompanion’ [52]. Images were elaborated with Adobe Illustrator.

## Results

### Strain Selection and Whole Genome Sequencing

A total of 107 microbial strains were isolated from the gut of the honeybees, but only a subset of 22 strains were identified as both fast-growing or strong substrate acidifiers (data not shown) and further processed for antimicrobial activities and bacteriocin isolation. The PFGE profile of the most representative strains is reported in Supplementary Figure S1. Sanger 16S rRNA sequencing data are reported in Table S3. Whole-genome sequencing results are reported in Table 1, as well as coverage and genome size.

**Table 1:** List of microbial strains sequenced in this work, their coverage, number of contigs above 500 bp, total genome length, GC (%), and GenBank accession number

Strain	Coverage	Contigs	Genome (bp)	GC (%)	Accession
*Bifidobacterium asteroides* C3	35.95	29	2,265,840	59.69	SAMN25059479
*Bifidobacterium coryneforme* C155	35.46	261	3,249,263	49.46	SAMN25059481
*Bifidobacterium indicum* C449	32.42	67	2,189,661	61.15	SAMN25059480
*Lactobacillus melliventris* Dan2	51.07	19	1,979,955	36.26	SCME00000000
*Lactobacillus kullabergensis* Dan23	96.31	33	2,105,329	35.74	SCMD00000000
*Lactobacillus kunkeei* Dan39	38.20	21	1,537,641	36.47	SCMC00000000
*Lactobacillus kimbladii *Dan47	36.50	23	1,911,448	35.67	SCMB00000000
*Lactobacillus apis* Dan63	115.77	17	1,871,693	36.85	SCMA00000000
*Lactobacillus helsingborgensis* Dan70	89.69	17	1,972,099	36.45	SCLZ00000000
*Lactobacillus plantarum *Dan91	120.92	36	3,278,303	44.39	SCLY00000000
*Lactobacillus johnsonii* Dan92	37.84	21	5,359,172	40.92	SAMN25059482

### qPCR Results

qPCR on Eubacteria (slope 3.62, intercept 38.31, and R^2^ 0.99) evidenced a total bacteria load ranging from Log 8.00 rRNA copies/intestine in [FC] group to Log 8.40 rRNA copies/intestine in [P]. Total bacteria were significantly higher in [P] group when compared to [C] and [FC] (*p* <0.05 and *p*<0.01, respectively), whereas all other comparisons were not significant. *Serratia* counts (slope 3.7, intercept 42.7, and R^2^ 0.99) were significantly higher (*p*<0.01) in [N] group reaching Log 5.4 CFU/intestine, when compared to the other experimental conditions that were in the range Log 4–4.2 CFU/intestine.

### NGS Results and Biodiversity Indices

**Fig. 2:**
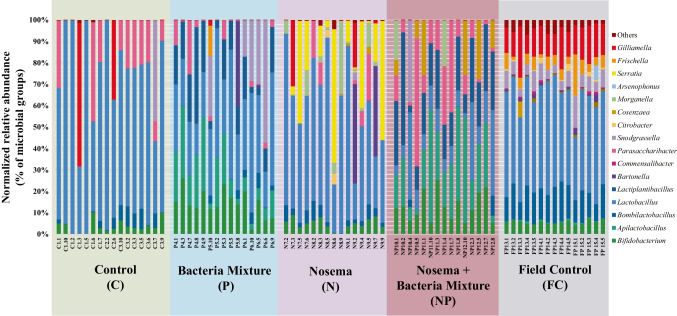
NGS relative abundance normalized with qPCR on total bacteria according to Baffoni et al. [24] (normalized relative abundance). Bar chart reporting the major cumulated microbial genera per sample and experimental condition: [C] honeybees fed with sugar syrup; (P) honeybees fed with sugar syrup enriched with BB mixture; [N] honeybees fed with sugar syrup and infected with *N. ceranae*; [NP] honeybees fed with sugar syrup, enriched with BB mixture and infected with *N. ceranae*; [FC] honeybees reared in field condition with sugar syrup supply as in Alberoni et al. [39]. The OTUs below 0.1% are grouped in the category named “Others”

A total of 60 samples (1 sampling time (at day 9) $\times$ 4 experimental conditions [C, P, N, NP] $\times$ 15 replicates for each condition obtained from single guts) were subjected to NGS analysis on Illumina MiSeq platform. Data related to FC group were retrieved from Alberoni et al. [39], in which the sampled honeybees had a similar age to those used in the present work. About 10 million raw reads were obtained from sequencing, 8.3 million of which passed the quality control and the chimera check analysis with an average of 69k joint reads per sample (ranging from 40,466 to 83,751 joint reads). For statistical analysis, samples were rarefied at 40,441 reads, a value obtained excluding one replicate (P4_2) because of low sequencing coverage. The taxonomical assignment of the 59 samples on the new taxonomical database produced 12,127 OTUs at 97% similarity based on SILVA v132 database. The obtained NGS data at genus level are reported in Table S5 while Fig. 2 reports relative abundance at genus level per replicate. Figure 2 clearly shows that the most affected gut microbial genera in caged honeybees were *Gilliamella*, *Frischella* and *Snodgrassella*, strongly reduced compared to [FC]. Moreover, *Lactobacillus* were dominant in both treated and untreated experimental caged conditions. Within Lactobacillaceae, the genus *Apilactobacillus* was dominant in experimental groups treated with the bacterial mixture. $\alpha$-diversity indices (Chao1, Observed_OTU, and PD_whole tree, see Table S6) showed that, in general, [P] group had higher values for Chao1 and Observed_OTU indexes and [NP] lower values compared to the other treatments. No differences between [C] and [FC] were registered for Chao1 index. [C] group had a significantly lower value compared to [P] and [N] (*p*<0.01, *p*<0.05), while no difference was highlighted between [C] and [NP]. On the other hand, [FC] registered a significantly higher value only compared to NP (*p*<0.05). The group infected and administered with beneficial microorganisms [NP] showed a Chao1 index significantly lower with respect to [P] (*p*<0.01) and [N] (*p*<0.01). The same trend can be underlined for Observed_OTU index, whose values resulted non-normal and heteroscedastic. No differences were recorded between [C] and [FC]. The [C] group had significantly lower OTU with respect to [P] (*p*<0.05) and [N] (*p*<0.05) but is not significantly different compared to [NP]. The field control [FC], as [C], is significantly lower compared to [P] (*p*<0.01) and [N] (*p*<0.01) and not significantly different compared to [NP]. The [NP] group had the lowest value of Observed_OTU index which was significant compared to [P] and [N] (*p*<0.01) showing index values over 1000. A different trend can be appreciated for PD_WT index because values were comparable among groups and only the laboratory control [C], which had the lowest score, was significant compared to the [P] group showing the highest score (*p*<0.01).

#### The Gut Microbiota of Caged Honeybees in the Different Experimental Conditions

**Fig. 3:**
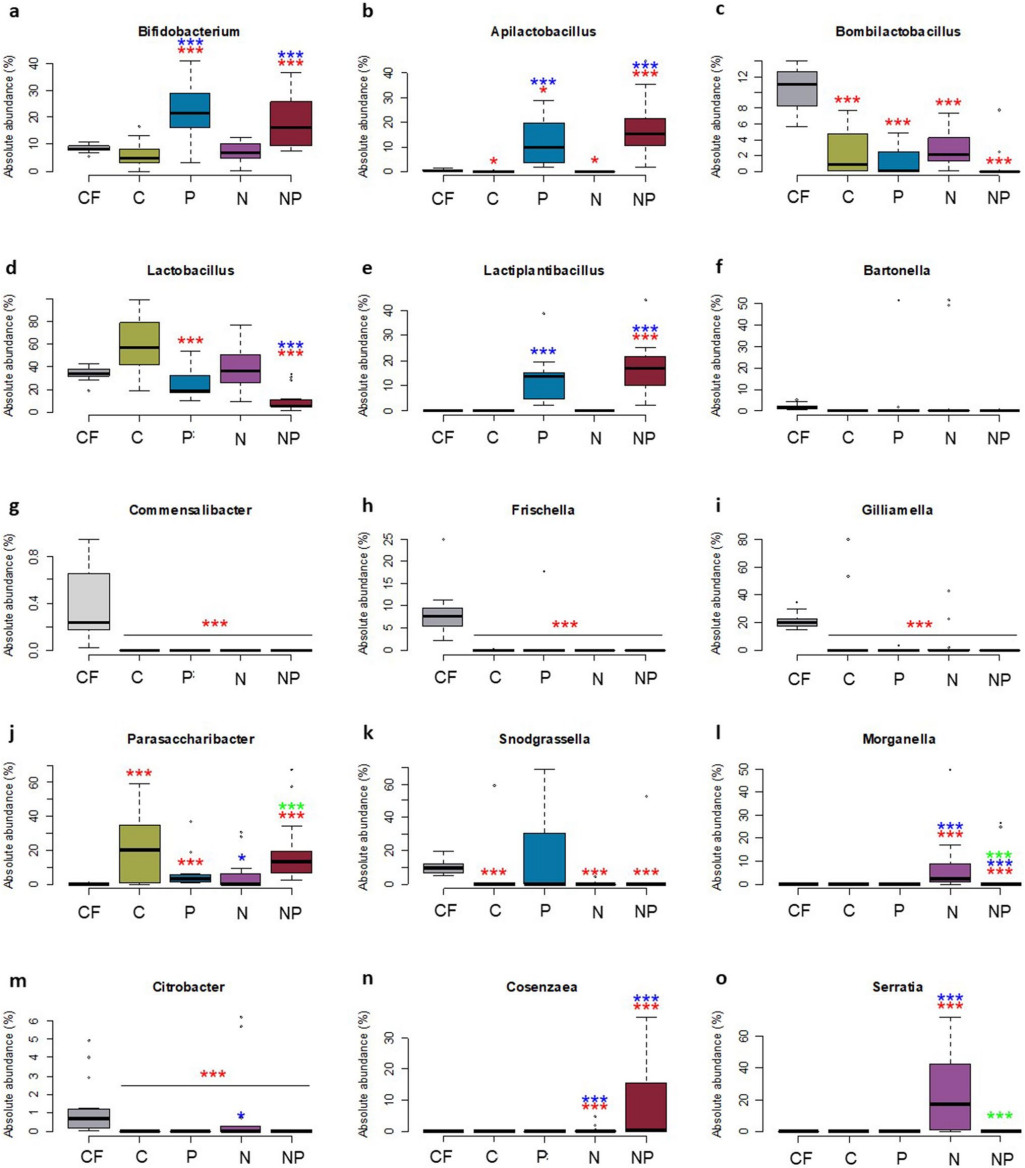
Boxplot reporting NGS relative abundance normalized with qPCR on total bacteria (normalized absolute abundance) at genus level of the most abundant taxa detected. [C] honeybees fed with sugar syrup; [P] honeybees fed with sugar syrup enriched with BB mixture; [N] honeybees fed with sugar syrup and infected with * N. ceranae*; [NP] honeybees fed with sugar syrup enriched with BB mixture, and infected with * N. ceranae*. [FC] honeybees reared in field condition with sugar syrup supply as in [39]. Significant pairwise comparisons *p<0.05; ***p<0.01. Asterisks in red refer to comparison of [FC] *vs* [C], [P], [N] and [NP]. Asterisks in blue refer to comparison of [C] *vs* [P], [N] and [NP]. Asterisks in green refer to the comparison of [N] *vs* [NP]

The gut microbial taxa showed significant shifts between the experimental conditions at the genus level as shown in Fig. 3A–O. When compared to [C], all the other cage experimental conditions showed a significant increase in *Apilactibacillus* (from average 0.00008% in [C] to 12.01% in [P] and 17.41% in [NP], Fig. 3B), *Bifidobacterium* (from average 5.84% in [C] to 22.30% in [P] and 18.53% in [NP], Fig. 3A), and *Lactiplantibacillus* (from average 0.00005% in [C] to 12.41% in [P] and 17.02% in [NP], Fig. 3E) (*p*<0.01). Conversely, *Parasaccharibacter* significantly decreased from average 20.27% of [C] to 5.66% of [N] (*p*<0.05, Fig. 3J), also *Lactobacillus* showed a general decrease in [N, P] and [NP] but only the latest was significant (59.20% in [C] *vs* 10.70% in [NP], *p*<0.01, Fig. 3D). *Bombilactobacillus* did not show significant variations (Fig. 3C), whereas the core genera *Commensalibacter*, *Bartonella*, *Gilliamella*, and *Frischella* were absent from almost all the honeybee samples with sporadic colonization or strongly underrepresented (Fig. 3F–I). *Snodgrassella* was completely absent from [C, N] and [NP] and it was detected only in few samples belonging to [P] in which 4 out of 15 individuals were strongly colonized by this genus belonging to the core genera (Fig. 3K). However, when [P] was compared with [C], the difference was not significant. Opportunistic commensal bacteria increased significantly only in experimental conditions with *N. ceranae*-infected honeybees. *Morganella* significantly increased from 0.000004% in [C] to 3.42% in [N] and below the limit of detection (LOD) of 10^-7^ in [NP] (*p*<0.01, Fig. 3L), *Citrobacter* from 0.000005% in [C] to average 0.87% in [N] (*p*<0.05, Fig. 3M), and *Cosenzaea* from average 0.00001% in [C] to 0.51% in [N] and 9.80% in [NP] (*p*<0.01, Fig. 3N). Finally, the pathogenic genus *Serratia* strongly increased from 0.00004% in [C] to 24.25% in [N] (*p*<0.01, Fig. 3O).

#### Comparison of Gut Microbiota of Caged Honeybees with Respect to Bees Reared in Hives

The gut microbiota composition of honeybees in field condition showed major differences when compared to the caged honeybees. In [FC] honeybees, core microbial groups such as *Commensalibacter* (0.39%), *Frischella* (8.34%), and *Gilliamella* (21.03%) were significantly higher when compared to all cage experimental conditions [C, P, N, NP] (*p*<0.01) that showed values close to or equal to 0.00%. *Lactobacillus* average abundance resulted in 34.4% in [FC] while it was significantly lower in [P] and [NP] (respectively 26.6% and 10.7%; *p*<0.01). Following the same trend, also *Bombilactobacillus*, with an average of 10.49% in [FC], is significantly lower with respect to [C] (2.48%), [N] (2.74%), [NP] (0.68%), and [P] (1.2%; all *p*<0.01). *Snodgrassella* showed an average population of 9.89% in [FC] that was significantly higher than [C] (3.70%), [N] (0.32%) and [NP] (3.50%; all comparisons *p*<0.01); on the contrary, *Snodgrassella* showed an average of 14.13% in [P], even if not significant compared to [FC]. In caged honeybees, higher percentages were detected for the genera *Apilactibacillus* (from an average of 0.38% in [FC] to 12.01% in [P] and 17.41% in [NP], *p*<0.01), *Bifidobacterium* (from an average of 8.57% in [FC] to 22.30% in [P] and 18.53% in [NP], *p*<0.01), and *Lactiplantibacillus* (from below LOD in [FC] to 17.02% in [NP], *p*<0.01). *Parasaccharibacter* showed a very low amount (0.01%) in [FC], while in caged honeybees high values were recovered (20.27% in [C], 5.66% in [N], 18.80% in [NP], and 6.20% in [P]; *p*<0.01). *Bartonella* did not show significant variations in the different experimental conditions. Finally, other opportunistic commensal bacteria like *Cosenzaea*, *Morganella*, and *Serratia* were below LOD in [FC], and significantly higher only in cage experimental conditions. An exception to this was represented by *Citrobacter* which showed 1.23% in [FC] but the percentage was significantly lower in cage conditions (0.87% in [N], below LOD in [P], 0.00005% in [C], and 0.008%[NP], all *p*<0.01).

#### The Gut Microbial Community in the Presence of *N. ceranae*

The comparison of [N] *vs* [NP] also showed a number of significant differences. Excluding the genus *Bifidobacterium* and the family Lactobacillaceae, whose differences are driven by the feed supplement, the main variations are represented by *Parasaccharibacter*, *Morganella*, and *Serratia* (*p*<0.01). All these genera were clearly more abundant in [N].

#### PCA Analysis

**Fig. 4:**
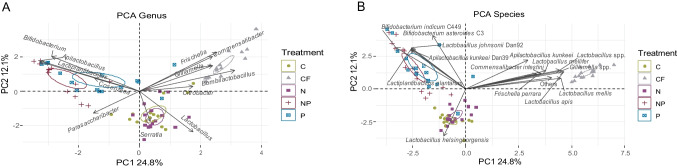
PCA. (A) PCA performed with 12 taxa at genus level. (B) PCA performed with 15 taxa at species level. Confidence ellipses are shown in the graphs

The PCA of the dataset at genus and species levels explained about 37% of the variability considering together PC1 and PC2. [FC] group is clearly separated along PC1 from all the other experimental conditions (Fig. 4A), a separation mainly driven by core bacterial taxa like *Frischella*, *Gilliamella*, and *Bombilactobacillus* and non-core taxa *Citrobacter* and *Commensalibacter*; while at species level, 3 Lactobacillaceae drivers were identified for [FC] group: *Apilactobacillus kunkeei*, *Bombilactobacillus mellis*, *Lactobacillus apis* (Fig. 4B). Along PC1 [C] and [N] experimental conditions clustered together as well as [P] and [NP], these two clusters are quite separated. Along PC2, the separation of [C]-[N] and [P]-[NP] is clearer, while [P]-[NP] are at the same height as [FC]. It can be speculated that PC1 is able to differentiate caged and not caged honeybees, while PC2 seems to differentiate stressed/not stressed insects considering that, even if caged [NP] and [P] groups are separated from [N] and [C] because supplementation may have an effect, mitigating cage conditions. [C] and [N], on the other hand, were stressed by cage condition without beneficial bacterial supplementation and also with infection of *N. ceranae*. In the [C] and [N] clusters, *Parasaccharibacter* and *Serratia* at the genus level, and *Lactobacillus helsingborgensis* at species level seemed to be the major drivers.

#### Evaluation of the 16S rRNA Gene Database Specific for Honeybees

The designed database was named InsectGene and its validation showed a successful taxonomical classification of over 99.9% of the representative OTUs. When the taxonomical assignment of OTUs performed with InsectGene was compared with that obtained with SILVA v132, no significant differences in the output results were noted at the genus level (Level 6 - genus) with the exception of *Commensalibacter* and *Parasaccharibacter*. On the other hand, taxonomical assignment at species level (Level 7 - species) of representative OTUs was much more accurate and reliable, especially in detecting the majority of strains supplemented in the bacterial mixture. The database was not capable of discriminating *Bifidobacterium asteroides* strains. Finally, the bioinformatic pipeline performed on a personal computer (Intel I-7 processor, 8Gb RAM) lasted 3.5 days for SILVA v132 whereas with InsectGene database was completed within 1 day. The total number of joint reads for each sample and the joint reads number attributed at genus level using both Silva v132 and InsectGene database are reported in Table S7.

#### Metabolic Pathways Identified in the Selected Strains

**Fig. 5:**
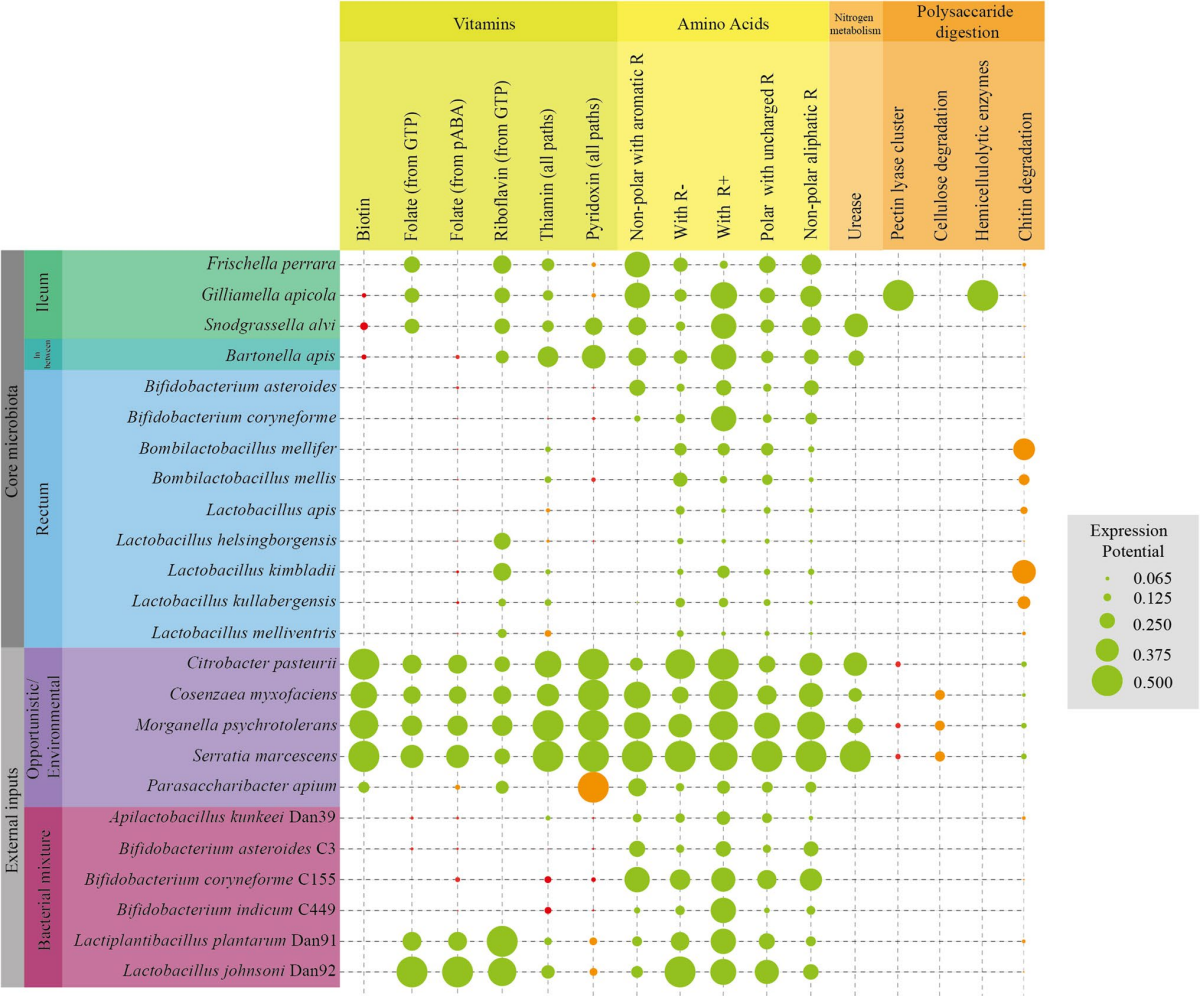
Functionality Dot Plot. The Dot-Plot represents the presumptive functionality of the microbial strains populating the microbiomes in this study, detected by NGS. Dot colours: Green represents complete metabolic clusters; orange represents incomplete metabolic clusters or complete presence in a strain and absence in other strains of the same species; red circle represents incomplete (nonfunctional) clusters but remarks the presence of some genes. If genes related to the cluster in the analysis are not detected, there are no circles reported. The circle sizes represent the number of genes detected, per metabolic category proportioned for every cluster

Genome analysis (Table S8) showed the presence of gene clusters for the biosynthesis of vitamins and amino acids, urea degradation, and polysaccharides hydrolysis (Fig. 5). Identified vitamin pathways were thiamine (B_1_), riboflavin (B_2_), pyridoxine (B_6_ or Y), biotin (B_7_), and folate (B_9_ or M). Functional clusters for thiamine synthesis were found in all strains except *Bifidobacterium*, but, within $\gamma$-proteobacteria, *Citrobacter*, *Cosenzaea*, *Morganella*, and *Serratia* (see Section “2.8” and “3.3”) had the most complete pathways for purine metabolism (*thiC*, EC2.7.1.49, EC2.7.4.7, EC2.5.1.3, and EC2.7.1.89) but also for tyrosine (starting from *thiG*) and steroids biosynthesis (starting from *thiH*). Lactobacillaceae were found capable of producing thiamine only from glycolysis intermediates (EC2.7.1.50 and EC2.5.1.3), whereas the remaining $\gamma$-proteobacteria (*Frischella*, *Gilliamella*, and *Snodgrassella*) showed complete pathways dedicated to tyrosine biosynthesis. Complete riboflavin synthesis pathway was detected in all the $\gamma$-proteobacteria taken into consideration and in *Citrobacter* (EC3.5.4.25, EC3.5.4.26, EC1.1.1.193, EC2.5.1.78, EC2.5.1.9, and EC4.1.99.12). Also, some *Lactobacillus* showed the presence of this biosynthetic pathway, such as *Lactobacillus helsingborgensis*, *L. kullabergensis*, *L. kimbladii*, *L. johnsonii*, and *Lactiplantibacilus plantarum*, but it is a sporadic presence within *Lactobacillus*. Pyridoxine biosynthesis pathway was mainly shown in non-core $\gamma$-proteobacteria and *Citrobacter*, a pathway active from D-erythrose 4-phosphate (EC1.2.1.72, EC1.1.1.290, EC2.6.1.52, EC1.1.1.262, EC2.6.99.2, EC1.4.3.5, EC2.7.1.35). *Bartonella* and *Snodgrassella* showed the same pathway but starting from 2-oxo-3-hydroxy-4-phosphobutanoate (after EC1.1.1.290). Pyridoxal 5’-phosphate synthase (EC1.4.3.5) is an enzyme detected in almost all strains considered, including Lactobacillaceae and *Bifidobacterium*. Only *Lactobacillus apis* hma11, *L. kullabergensis* Dan23, and *L. melliventris* Hma8 did not show any gene related to pyridoxine. Biotin biosynthesis cluster was identified only in *Citrobacter*, *Cosenzaea*, *Morganella*, and *Serratia* (EC2.3.1.47, EC2.6.1.62, EC6.3.3.3, EC2.8.1.6). *Gilliamella* and *Snodgrassella* showed incomplete clusters, whereas Lactobacillaceae, *Bartonella*, and *Bifidobacteria* did not show the biotin production cluster in any of the studied strains. Finally, folate biosynthesis pathways were identified with two different precursors: guanosine triphosphate (GTP) and *p*-aminobenzoic acid (*p*-ABA). All $\gamma$-proteobacteria, *L. johnsoni* Dan92 and *L. plantarum* Dan91, showed complete GTP clusters (EC3.5.4.16, EC3.1.3.1, EC4.1.2.25, EC3.6.1.-, EC2.7.6.3, EC2.5.1.15, EC6.3.2.12/17, and EC1.5.1.3; p-ABA path EC2.6.1.85 and EC4.1.3.38). Core Lactobacillaceae honeybees and *Bifidobacterium* species showed an incomplete pathway starting from 7,8-dihydropteroate to folate (EC6.3.2.12/17 and EC1.5.1.3). Amino acid (AA) biosynthetic pathways were highly represented for all the AA groups (non-polar with aromatic R, with positively charged R, with negatively charged R, polar with uncharged R, non-polar with aliphatic R) among all $\gamma$-proteobacteria, *Bartonella* and *Citrobacter*. All Lactobacillaceae possessed a reduced ability to produce AA lacking a number of metabolic pathways for single AA (e.g. isoleucine, valine, histidine, phenylalanine, tyrosine and cysteine), or for the reduced gene number present in functional pathways when compared to $\gamma$-proteobacteria, *Bartonella* and *Citrobacter*. The only exception to this is represented by *L. plantarum* Dan91 and *L. johnsoni* Dan92 which showed consistent AA gene sets. Among the honeybee core genera, the urease cluster (EC3.5.1.5) was detected only in *Bartonella* and *Snodgrassella* whereas in environmental and opportunistic bacteria it was always present. The pectin lyase cluster was found present only in *Gilliamella*. Incomplete clusters for cellulose degradation (EC3.2.1.4 Endoglucanase) were found in *Cosenzaea*, *Morganella*, and *Serratia*. Beta-glucosidase enzyme cluster (EC3.2.1.86; EC3.2.1.21 and EC3.2.1.20), whose function is promiscuous in the polysaccharide’s digestion, was found present in multiple gene copies in all the species analysed, except for *Bifidobacterium asteroides*, *Snodgrasella*, and *Bartonella* species. Complete clusters of hemicellulolytic enzymes (EC3.2.1.15, EC3.2.1.40, and EC3.2.1.52) were found only in *Gilliamella*. Finally, chitinases were found to be present in all the analysed species, with particular relevance in Lactobacillaceae in which some strains showed the presence of 12–16 genes of the sole chitinase (EC3.2.1.14), as well as few beta-N-acetylhexosaminidase (EC3.2.1.52) and N-acetyl-D-glucosamine 6-phosphotransferase (EC2.7.1.59) genes.

#### Comparison of Metabolic Pathways in the Different Experimental Conditions

**Fig. 6:**
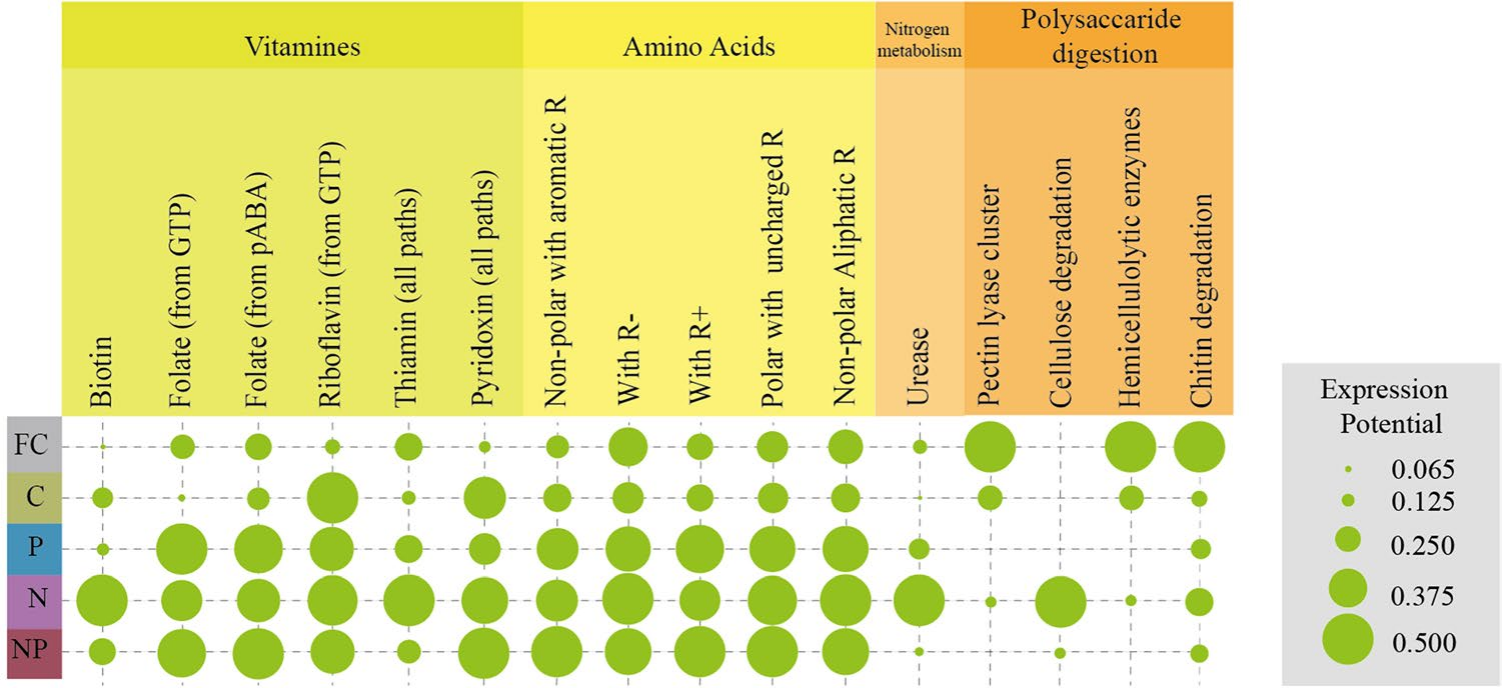
Cumulative functionality Dot-Plot in the considered experimental conditions. The Dot-Plot represents the presumptive and cumulative functionality of the microbial strains populating the microbiomes in this study, per experimental condition. If genes related to the cluster in analysis are not detected, there are no circles reported. The circle sizes represent the number of genes detected, per metabolic category proportioned for every cluster

Pairwise comparison of the genetic potential revealed significant variations for the majority of the experimental conditions (Fig. 6, Table S9). Biotin genes were significantly enriched in [N] and [NP] *vs* [FC] (*p*<0.01). Genes for folate production were also enriched in groups where any treatment was applied ([N], [P], and [NP]) compared to [C] (*p*<0.01). Riboflavin genes significantly increased in all cage-based experimental conditions compared to [FC] (*p*<0.01). Aminoacid biosynthesis potential increased significantly in groups [P, N] and [NP] when compared to [C] or [FC]. Potential urease activity was higher in [P, N] and [NP] when compared to [C] but also when comparing [N] *vs* [NP]. Polysaccharide digestion was significantly lower only in [NP] condition when compared to [C] or [FC]. Finally, hemicellulose and chitin degradation abilities were much higher in field [FC] than in cage conditions. However, when the data were analysed in aggregate form, and [FC] compared to [C], [P], [N] and [NP] or [C] compared to [P], [N] and [NP], the CramerV model, measuring the differential gene expression potential, evidenced a medium-low biological relevance of any comparison ([FC] *vs* [C] CramerV = 0.30; [FC] *vs* P = 0.25; [FC] *vs* [N] = 0.29; [FC] *vs* [NP] = 0.30; [C] *vs* [P] = 0.24; [C] *vs* [N] = 0.25; [C] *vs* [NP] = 0.21).

## Discussion

Research in the last decade has contributed to the elucidation of several gut microbiota functions in honeybees. These functions span from the digestion of high-protein feeds like pollen [53], to the metabolization of indigestible or toxic sugars like mannose and xylose [54] and the production of organic acids [53]. This work is focused on understanding whether the rearing of honeybees in cages, as frequently happens in laboratory experimental assays, may influence the gut microbiota acquisition and functions, also considering the presence of pathogens and the administration of bacteria as a feed supplement. The comparison of the microbiota of caged honeybees with that of in-field hive conditions [FC] highlighted that proper development of the intestinal microbiota is hindered in cage conditions. This dysbiotic condition, defined by Maes et al. [55] as “shifts in bacterial community composition, capable to lower the colonization resistance of the gut to intrinsic pathogens” may favor *N. ceranae* development or hinder the action of food additives tested for its control, such as the mixture of microorganisms. Caged honeybees, picked from brood before the acquisition of the intestinal microbiota, showed, after 9 days, that several taxa were capable of colonizing the gut despite the lack of contact with adult honeybees or hive environment. In particular, the genera and species belonging to the Lactobacillaceae family and *Bifidobacterium* seemed to be acquired even without horizontal transmission. The core $\gamma$-proteobacteria (*Frischella*, *Gilliamella*, *Snodgrasella*) were found only in few individuals in cage experimental conditions. Therefore, these genera were hardly transmitted by trophallaxis among the members of each cage. It can be speculated that the low number of individuals per cage did not allow the development of social cohesion that normally leads to the activation of trophallaxis and coprophagy mechanisms. $\alpha$-diversity analysis showed that the treated but non-infected group [P] displayed the highest values for all indexes: Chao1, which gives importance to rare OTUs, Observed_OTU, underlining the increased richness of the [P] group and PD_whole_tree, showing a small increase in phylogenetic diversity for treated honeybees. PD_whole_tree in the different groups is however comparable, evidencing that taxa related to honeybee gut are clearly defined, with low perturbations in the different experimental conditions. On the other hand, the treated and infected group [NP] displayed the lowest number of observed OTU and the lowest Chao1 index. Comparing [FC] *vs* [C], no substantial differences can be observed; however, the analysis shows that any perturbation (like microorganisms administration or infection) favors a shift that modifies the balance of the gut taxa. The results obtained clearly showed that *N. ceranae* contributed to the colonization of opportunistic bacteria. These were not derived from *N. ceranae* artificial infection, considering that the *N. ceranae* inoculum was purified by Percoll sedimentation  [21] and that other genera typical of the honeybee gut microbiota were not co-inoculated with the microsporidium in caged honeybees, such the core $\gamma$-proteobacteria. On the contrary, opportunistic bacteria typical of the hive environment such as *Citrobacter*, *Cosenzaea*, *Morganella *, and *Serratia* [53, 54, 56] may derive from the chewing of the operculum during eclosure. However, these genera were detected only when *N. ceranae* was present, and with relevant percentages when microorganisms were not administered. Indeed, in the [NP] samples, the proliferation of opportunistic bacteria was significantly reduced, especially in the case of *Serratia*. Therefore, opportunistic pathogens seemed to take advantage of a compromised gut microbiota in agreement with Brown et al. [59]. The absolute quantification of *Serratia* confirmed its significant increase in [N] experimental condition. Our data, therefore, support the hypothesis that the bacterial formulation supplied (mainly Lactobacillaceae and Bifidobacteriaceae) is effective to control the proliferation of opportunistic bacteria such as *Serratia*.

The newly developed InsectGene database was found to be efficient in analysing the honeybee gut raw data, also overcoming some limitations of the Silva databases. In particular, the assignment at species level was improved for the insects’ gut microbiome, as InsectGene can be easily upgraded by the users according to the latest taxonomical updates (*e.g.* with newly described species [36, 60]). Moreover, the database can be upgraded also for NGS data analysis of microbial niches based on different treatments, as in our case was done after administration of the microbial based feed supplements. Finally, InsectGene database showed an efficient use of computational resources and time saving. However, an analysis based on a tailored database can be applied only when taxonomical groups inhabiting a target environment are well known and do not vary considerably upon treatment applications.

The accurate taxonomic identification of OTUs allowed a proper functional analysis of the gut microbiota. Gut microbiome functionality is hard to analyse *in silico* because we do not know if the detected genes are effectively expressed and how much they are expressed in real environmental conditions (*e.g.* extracellular enzymes, antimicrobial substances, vitamins, amino acids). However, manual curation of the different steps of the studied pathways may help in the determination of the completeness of a target metabolic pathway, considering that many enzymes are shared among different pathways. In the present study, an accurate focus was addressed on vitamins that insects are unable to synthesize, such as several B vitamins [61], with rare exceptions for B_5_ (pantothenate) and B_7_ (biotin) in certain insects. Therefore, these vitamins must be acquired from the environment or from the gut microbiota. Among the most abundant vitamins detected in most experimental condition, folic acid genes are highly enriched not only in [P, NP] and [N], but also in [FC]. In honeybees, a moderate folic acid dose (0.05 mg/kg) has been found to improve newborn queen bees’ performances [62], but an excess negatively affects newborn queens, supporting that an imbalance of some biochemical compounds can strongly impact the host physiology. Surprisingly, the main producers of folate were not *Bifidobacterium* strains which are well-known folate producers [63], but *Gilliamella*, *Frischella*, and *Snodgrassella* strains and environmental bacteria, all synthesizing folic acid from GTP only. Supplemented Lactobacillaceae also contributed to folate supply from the p-ABA pathway but interestingly both commensal and supplemented bifidobacteria analysed did not show any active pathway. Thiamin, abundant in [N] group, is known to support, together with riboflavin, hypo-pharyngeal glands development and honeybee longevity [64]. Pyridoxine, whose expression potential was high in all cage experimental conditions and low in [FC], is able to support larva pupation [65]. As for vitamins, there are essential amino acids that honeybees must acquire from the environment (pollen and nectar) [66] but presumably also from the gut microbiome. Interestingly, some studies report the amino acid release of bacteria into a nitrogen-free medium. Matteuzzi et al. [67] showed that strains of bifidobacteria can release in broth medium considerable amounts of amino acids (*e.g.* up to 150 mg/L of threonine from *Bifidobacterium bifidum*). A similar mechanism can be hypothesized in the honeybee gut, where the microbial community is capable of producing all the amino acids necessary for honeybees, in [P], [N] and [NP]. Regarding the nitrogen metabolism, urea, as waste product, is typically harvested by Malpighian tubes and condensed in the digestive tract. *Snodgrassella* and *Bartonella* and environmental bacteria were found to be able to degrade urea into ammonia and CO_2_; however, related genes seemed to be enriched only in [N] condition. The capability to break down urea confirms a gut symbiont-driven nitrogen recycling mechanism in honeybees. This mechanism was firstly validated in *Melolontha hippocastani* by Alonso-Pernas et al. [68] in which gut microbiomes were capable of mediating urea breakdown and ammonia re-use. Pollen utilization requires pectin, hemicelluloses, and cellulose degradation. Interestingly, both pectin and hemicellulose degradation gene complexes were found only in *Gilliamella*, in agreement with Zheng et al. [69]. Therefore, the relevant presence of environmental and opportunistic bacteria in [N] did not improve the pollen degradation capability, with the exception of some cellulose-degrading enzymes, related to an incomplete pathway. Finally, chitinases may be secreted by insects in order to control the proliferation of pathogenic fungi [70]. A similar role of the gut microbiome might be hypothesized, also in consideration of the very high copy number of chitinases (up to 16 per strain) detected in some Lactobacillaceae. This may help Lactobacillaceae and Bifidobacteriaceae to outcompete yeast proliferation in an environment attractive also for fungi. When samples from field condition are compared to caged ones, only [C] and [FC] showed similar metabolic profiles. Surprisingly, the potential synthesis of vitamins and amino acids, as well as urea degradation, is much higher in experimental conditions either with *N. ceranae* or with the microbial mixture [N, NP, P], showing that *N. ceranae* infection seemed to improve the number of essential compounds potentially available in the gut. Urea and cellulose degradation are boosted in [N]. In natural infection conditions, this leads to an improved carbon digestion or nitrogen metabolism. On the whole, the functionality expressed by the gut microbiome seemed to improve when *N. ceranae* is present.

## Conclusion

This work highlighted that *N. ceranae* can favor the development of non-core bacteria, contributing to gut dysbiosis in newly eclosed caged honeybees. In these conditions, honeybees appear to be unable to acquire core $\gamma$-proteobacteria, and this should be considered in the design of cage-based tests. This work also showed that a mixture of Lactobacillaceae and bifidobacteria prevented the colonization of environmental potentially harmful bacteria in co-infection with *N. ceranae*. Finally, it was found that the production of vitamins and amino acids, as well as urea degradation and cellulose digestion, improved when *N. ceranae* was present, or when the bacteria mixture was supplemented, in agreement with its parasitic behavior that can alter host physiology and behavior in order to maintain a more favorable environment for its reproduction.

## Supplementary Information

ESM 1 (JPG 1299 kb)

ESM 2 (XLSX 79 kb)

## Data Availability

16S rRNA sequence data have been submitted to the NCBI repository Sequence Read Archive (SRA) under the Bio project nÂ° PRJNA669646, accession numbers SAMN16442367 - SAMN16442371; SAMN16442379 - SAMN16442383; SAMN16442385 - SAMN16442389 and SAMN25084972 - SAMN25085032. The Whole Genome Shotgun can be found under the BioProject number PRJNA515431, BioSamples accession number SAMN10754954 - SAMN10754960 and SAMN25059479 - SAMN25059482. Supplementary data, including Excel files of elaborated data obtained from qPCR for target microbial groups and NGS data categorized at genus and species levels, are available on reasonable request from the corresponding author. InsectGene database repository:
