# Supplementary figures and images for: Alterations in the Microbiota of Caged Honeybees in the Presence of Nosema ceranae Infection and Related Changes in Functionality
Source: Microb Ecol. 2022 Jul 12;86(1):601–16. doi: 10.1007/s00248-022-02050-4 (PMC10293464; doi:10.1007/s00248-022-02050-4)

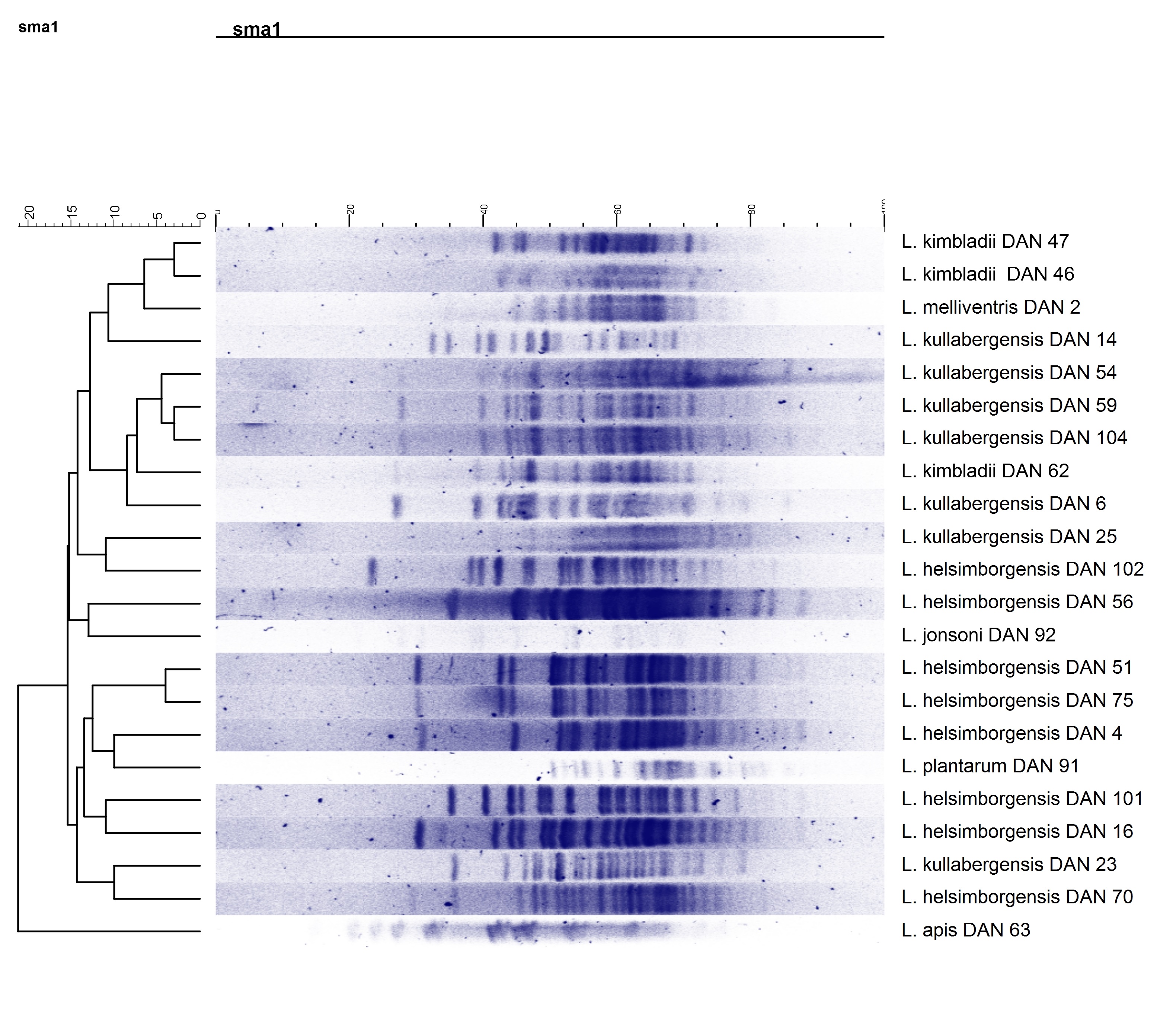

Supplement: Supplementary file 1 — (JPG 1299 kb) [file 248_2022_2050_MOESM1_ESM.jpg]
